# The influence of spatially heterogeneous anthropogenic change on bill size evolution in a coastal songbird

**DOI:** 10.1111/eva.13144

**Published:** 2020-10-21

**Authors:** Phred M. Benham, Rauri C. K. Bowie

**Affiliations:** ^1^ Museum of Vertebrate Zoology University of California Berkeley, Berkeley CA USA; ^2^ Department of Integrative Biology University of California Berkeley, Berkeley CA USA

**Keywords:** Anthropogenic change, bill morphology, California, evaporative water loss, museum specimens, Passerellidae, thermoregulation

## Abstract

Natural history collections provide an unparalleled resource for documenting population responses to past anthropogenic change. However, in many cases, traits measured on specimens may vary temporally in response to a number of different anthropogenic pressures or demographic processes. While teasing apart these different drivers is challenging, approaches that integrate analyses of spatial and temporal series of specimens can provide a robust framework for examining whether traits exhibit common responses to ecological variation in space and time. We applied this approach to analyze bill morphology variation in California Savannah Sparrows (*Passerculus sandwichensis*). We found that bill surface area increased in birds from higher salinity tidal marshes that are hotter and drier. Only the coastal subspecies, *alaudinus,* exhibited a significant increase in bill size through time. As with patterns of spatial variation, *alaudinus* populations occupying higher salinity tidal marshes that have become warmer and drier over the past century exhibited the greatest increases in bill surface area. We also found a significant negative correlation between bill surface area and total evaporative water loss (TEWL) and estimated that observed increases in bill size could result in a reduction of up to 16.2% in daily water losses. Together, these patterns of spatial and temporal variation in bill size were consistent with the hypothesis that larger bills are favored in freshwater‐limited environments as a mechanism of dissipating heat, reducing reliance on evaporative cooling, and increasing water conservation. With museum collections increasingly being leveraged to understand past responses to global change, this work highlights the importance of considering the influence of many different axes of anthropogenic change and of integrating spatial and temporal analyses to better understand the influence of specific human impacts on population change over time.

## 1 INTRODUCTION

Human activities such as urbanization and pollution have profoundly altered the planet, resulting in habitat loss, climate change, and other ecological alterations (IPBES, 2019). Such rampant, human‐mediated ecological change imposes novel pressures on natural populations that can lead to a range of organismal responses including adaptive change (Campbell‐Staton et al., 2020; Jain & Bradshaw, 1966; Oziolor et al., 2019; van’t Hof et al., 2016), distribution shifts (Crimmins et al., 2011; Rowe et al., 2015; Tingley et al., 2009, 2012), and extinction (Pimm et al., 2006; Schipper et al., 2008; Stuart et al., 2004). These wide‐ranging impacts make understanding how populations respond to different axes of anthropogenic change a pressing challenge (Moritz & Agudo, 2013). Accurately linking past population responses to specific anthropogenic pressures can inform predictions of future organismal responses, yet inferences about the impacts of anthropogenic change remain in many cases limited to analyses of samples collected long after human transformation of the landscape. The millions of specimens and associated data in the collections of natural history museums span broad spatial, temporal, and taxonomic scales and thus represent a potentially crucial resource for documenting change over time and discerning the specific anthropogenic drivers of observed change (Holmes, Hammond, et al., 2016, 2016; Lang et al., 2019; Meineke et al., 2019).

A growing catalog of empirical research using museum specimens now provides abundant evidence of temporal changes in distribution (Moritz et al., 2008; Rowe et al., 2015; Tingley et al., 2009, 2012), coloration (Mason & Unitt, 2018), phenology (Kiat et al., 2019), body size and shape (Gardner et al., 2011, 2019; Weeks et al., 2019), morphology (Assis et al., 2016; Campbell‐Tennant et al., 2015; Holmes, Hammond, et al., 2016, 2016; Kitano et al., 2008; Miller et al., 2018; Millien et al., 2017), diet (McMahon et al., 2019; Norris et al., 2007; Walsh et al., 2016), and genotypes (Bi et al., 2019) of many species. However, specimen‐based studies also show idiosyncratic responses across co‐distributed species or populations of the same species (Miller et al., 2018; Onley et al., 2020; Rowe et al., 2015; Salewski et al., 2014; Tingley et al., 2012). These differences in the magnitude and direction of temporal change may be explained by a number of potentially interacting factors. First, many phenotypic traits measured in museum specimens are involved in multiple functions critical to organismal performance and fitness. Thus, spatial–temporal variation in these characters may reflect the need to optimize performance in the face of multiple independent ecological pressures (Friedman et al., 2019). Second, patterns of temporal change in relevant ecological pressures may vary spatially (Senner et al., 2018; Tingley et al., 2012). Third, population demography (e.g., fluctuations in effective population size or changes in gene flow patterns) may also influence patterns of temporal variation (Grabenstein & Taylor, 2018). Finally, whether relevant standing genetic variation exists in a population for selection to act upon (Barrett & Schluter, 2008) and the degree to which traits are heritable or plastic (Teplitsky et al., 2008) may further shape observed patterns of phenotypic change through time. Although it would be difficult to address all of these possibilities in a single study, analyzing patterns of temporal change in museum specimens sampled from across a landscape of nonuniform anthropogenic change provides a potentially informative framework for disambiguating the relative influence of different forces on population change. Few studies to date have explicitly integrated the examination of spatial and temporal series of museum specimens using such a framework. To explore the efficacy of this approach, we analyzed the potential drivers of spatial and temporal variation in a multi‐functional trait that is readily measured on specimens: bird bills.

The dimensions of bird bills have generally been found to be highly heritable (Boag & Grant, 1978), but some evidence exists for their developmental (James, 1991) and seasonal plasticity (Greenberg et al., 2013). Given the primary function of bills for foraging, both spatial and temporal variations in bill morphology are often interpreted through the lens of diet and foraging ecology (Benkman, 2003; Boag & Grant, 1981; Bosse et al., 2017; Francis & Guralnick, 2010). However, climatic parameters may also contribute significantly to variation in bill morphology, as bird bills are heavily vascularized and can be used as thermoregulatory organs by actively regulating blood flow to the bill in response to temperature change (Greenberg et al., 2012; Hagan & Heath, 1980; Tattersall et al., 2009, 2017, 2018; van de Ven et al., 2016; van Vuuren et al., 2020). This functionality suggests that bird bills should vary in time and space in association with temperature variation, a finding which has been supported by research into a number of bird species (Campbell‐Tennant et al., 2015; Danner & Greenberg, 2015; Symonds & Tattersall, 2010). Birds also rely heavily on evaporative cooling for thermoregulation (Bicudo et al., 2010), and the influence of temperature on bill size variation could be further mediated by the influence of different selective pressures on evaporative water loss. First, evaporation will be less efficient at dissipating excess body heat in more humid environments (Gerson et al., 2014), and heat dissipation across a larger bill surface area may be favored in hotter and more humid conditions (van Gardner et al., 2016; Gardner et al., 2016; van de Ven et al., 2016). Second, increased heat dissipation across larger bills in environments with limited freshwater‐availability (e.g., tidal marshes) could reduce reliance on evaporative cooling and contribute to increased water conservation (Greenberg et al., 2012). These prior analyses of bill size variation indicate that temporal changes in bill size can reflect variations in demography (e.g., gene flow or drift), diet, and/or interactions between temperature and other climatic factors such as humidity, precipitation, and salinity.

**Figure 1:**
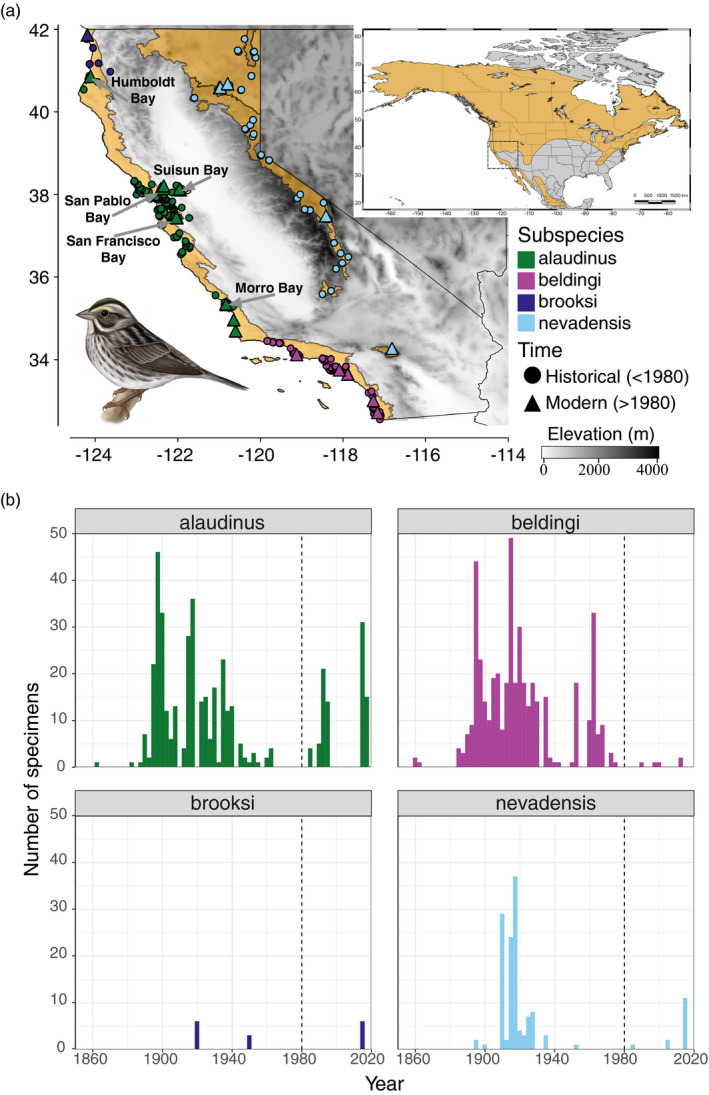
(a) California breeding distribution of the Savannah Sparrow (ocher). Circles represent historical (pre‐1980) sampling localities for specimens that were measured in the present study, and triangles represent modern (post‐1980) sampling localities. Different colors correspond to the four Savannah Sparrow subspecies that breed in California. Accompanying illustration is of the *P. s. beldingi* subspecies (artwork courtesy of: Jillian Nichol Ditner). Inset shows extent of the entire North American breeding distribution of the Savannah Sparrow in ocher with dotted box outlining main map of California populations. (b) Temporal distribution of specimens measured for each subspecies. Dashed line at 1980 represents the time point used to divide the dataset into historical and modern groups used for certain analyses

Here, we explore how these different factors may contribute to patterns of spatial and temporal variation in bill morphology across California populations of the Savannah Sparrow (*Passerculus sandwichensis*). Savannah Sparrows are one of the most widespread North American songbirds, with 17 subspecies breeding across a range of open habitats from Alaskan tundra to Guatemalan highlands (Figure 1a; Wheelwright & Rising, 2008). We focus on California populations due to the dense spatial and temporal sampling that has been conducted in California and the availability of large datasets on the state's climate, habitat, and environmental conditions (e.g., https://www.ecoatlas.org). Within California, four subspecies occur (Grinnell & Miller, 1944; Figure 1b). *Passerculus. sandwichensis brooksi* (hereafter *brooksi*) and *P. s. nevadensis* (hereafter *nevadensis*) are both migratory subspecies that occupy grasslands in extreme northwest and eastern California, respectively. *Passerculus sandwichensis alaudinus* (hereafter *alaudinus*) resides in both tidal marsh and coastal grassland habitats from Humboldt Bay (Humboldt County) south to Point Conception (Santa Barbara County). *Passerculus sandwichensis beldingi* (hereafter *beldingi*) is a year‐round resident of tidal marshes from Point Conception south into Baja California. *Beldingi* is estimated to have diverged from the other three subspecies more than 400,000 years ago, but little genetic structure exists among the other three subspecies (Benham & Cheviron, 2019). Previous studies also document ongoing gene flow among all subspecies and clinal variation in genetic diversity, with interior and northern populations exhibiting greater nucleotide diversity than southern coastal populations (Benham & Cheviron, 2020).

During the breeding season, California Savannah Sparrows are broadly distributed across climatic zones that range from cooler and wetter conditions in the northwest to hotter and drier conditions in the southeast (Grinnell & Miller, 1944). Variation in the salinity of habitats occupied by California Savannah Sparrows also imposes significant selective pressures on populations found in tidal marshes, where prior work has documented physiological, behavioral, and genomic divergence between tidal marsh and other California populations of the species (Benham & Cheviron, 2020; Cade & Bartholomew, 1959; Poulson & Bartholomew, 1962; Walsh et al., 2019). Tidal marsh populations of Savannah Sparrow, especially *beldingi*, also exhibit longer bills with larger surface areas than those of interior populations (Greenberg, et al., 2012; Grenier & Greenberg, 2005; Rising, 2001).

The past century has seen dramatic ecological change across California along a number of axes that could influence patterns of bill morphology in Savannah Sparrows, including the following: climate change (Cordero et al., 2011; Ficklin & Novick, 2017; Tingley et al., 2012); growth of the human population by over 38 million since 1900 (U.S. Census Bureau, 2019); widespread habitat loss from agriculture, urbanization, and industry (Takekawa et al., 2006); and changing plant communities due to both range shifts and invasive species (Crimmins et al., 2011; Takekawa et al., 2006; Thorne et al., 2008). While human‐mediated ecological change has impacted much of California, these anthropogenic pressures have not been of uniform severity across the state. Human population growth has primarily been concentrated in large metropolitan regions, such as San Francisco and Los Angeles, while many areas of the Sierra Nevada remain wilderness. Patterns of climate change also vary widely throughout the state. For example, maximum temperatures have increased the most in urban and agricultural areas, but have decreased along the immediate coast (LaDochy et al., 2007; Lebassi et al., 2009). Finally, agriculture, urbanization, and certain industries (e.g., salt evaporation mining) have collectively contributed to the loss of 75% of the state's coastal wetlands (Powell, 2006), including critical tidal marsh habitat for many Savannah Sparrow populations. However, while the loss of tidal marsh habitat approaches 90% in some estuaries (e.g., San Francisco Bay; Marshall & Dedrick, 1994), other estuaries have experienced the expansion of their tidal marsh habitat (e.g., Morro bay; Gerdes et al., 1974). The conversion of tidal marsh habitat to urban environments or salt mining operations has eliminated much of the available habitat for this species, but sparrows have also expanded their range into these novel habitats in areas where the marshlands have been diked for agriculture and pasture (Fitton, 2008).

These nonuniform patterns of temporal change in ecological parameters across California provide an opportunity to test predictions about the influence of different ecological drivers on bill size change. Specifically, we expect that the different ecological factors that contribute most to spatial variation in bill size will also exhibit the greatest magnitude of temporal change in bill size variation. To examine these observations further, we generated a large morphological dataset of California Savannah Sparrows to address the following questions: (a) Which environmental variables best explain variation in bill morphology across California populations of the Savannah Sparrow? (b) Does bill morphology vary significantly through time within any of the measured populations? (c) If so, what climatic, habitat, and demographic factors contribute to patterns of temporal variation in bill morphology? And (d) does temporal variation in bill morphology have any functional significance related to thermoregulation and water conservation?

## 2 METHODS

### 2.1 Sampling

#### 2.1.1 Morphological data collection and filtering

We measured bill length, bill width, bill depth, tarsus length, unflattened wing chord, and tail length in museum specimens and in live Savannah Sparrows sampled from throughout California between 1863 and 2017 (Figure 1b; Figure S1). Bills were measured from the anterior part of the nares. PMB performed all measurements using digital calipers with 0.01 mm precision. We calculated bill surface area using a formula for the lateral surface area of a cone (Equation 1) that has been frequently used to estimate total bill surface area in birds (LaBarbera et al., 2017).

(1) $$\frac{Billdepth+Billwidth}{4}*Billlength*\pi$$

We included only adult birds in the dataset and excluded individuals from the winter months whose wing‐length exceeded 70 mm. This threshold exceeded the distribution of resident birds collected during the breeding season and the various migratory subspecies that winter in coastal California largely have wing lengths exceeding 70 mm, though some overlap does exist (Pyle, 1997). Measurements were taken from 17 live birds captured at two localities (Morro Bay, San Luis Obispo Co.; and Grizzly Island Wildlife Management Area, Solano Co.). Protocols for capturing and measuring birds were approved by ﻿the University of Illinois, Urbana‐Champaign IACUC (protocol #: 13418), United States Fish & Wildlife Service (permit #: MB24360B), and California Department of Fish & Wildlife (permit #: SCP‐012913). We accounted for differences arising due to shrinkage in dried specimens (Winker, 1993) by estimating the percentage change in morphological characters between recently collected birds and measurements taken four years later from dried specimens of the same individuals. All downstream analyses were performed on a dataset with live birds corrected for the percent shrinkage. After the above filtering steps, the dataset included 949 specimens (Table 1; Figure 1b).

**Table 1:** Sample size of measured birds following filtering

Subspecies	Total adults	Historic (pre‐1980)		Modern (post‐1980)	
		male	female	male	female
*P. s. alaudinus*	526	265	179	66	16
*P. s. beldingi*	294	180	109	3	2
*P. s. brooksi*	14	5	3	6	0
*P. s. nevadensis*	110	63	33	12	2

#### 2.1.2 Climatic data collection

For each sampling locality, we collected data on temperature, precipitation, and humidity for each month from 1895 to 2018 from the PRISM climate database (http://www.prism.oregonstate.edu; Gibson et al., 2002). From the PRISM data, we estimated annual maximum temperature, annual minimum temperature, annual precipitation, maximum and minimum vapor pressure deficit, and mean dew point for each year between 1895 and 2018. For each specimen, we extracted values from each environmental parameter for the year and location of specimen collection.

#### 2.1.3 Environmental and urbanization data

To account for potential ecological differences associated with habitat differences (e.g., diet), each specimen was assigned to one of the 19 USDA Ecoregions as a proxy for habitat (Cleland et al., 2007). We collected salinity data from the California Environmental Data Exchange Network database (CEDEN; https://ceden.waterboards.ca.gov/AdvancedQueryTool), which includes hydrological parameters from sampling sites across California. For each sampling station, we calculated mean salinity from all samples taken. For specimens collected in landlocked counties, we used mean salinity from the entire county, as available water was typically <1 ppt throughout the county. In coastal areas, we estimated mean salinity from across all sampling stations within estuaries and nearby coastal sites where birds were sampled using the basic statistics analysis tool in QGIS 3.4 (https://www.qgis.org/en/site/). Secondly, we also included salinity data from water samples collected at the sites of capture for several of the sparrows included in this dataset (data from Benham & Cheviron, 2020).

Further, we collected data on a number of urbanization and habitat metrics for five coastal estuaries with sufficient modern and historic specimens (*n *≥ 5 for both periods). These coastal estuaries span the latitudinal distribution of *alaudinus* and include the following: Morro Bay, San Francisco Bay (south of San Francisco), San Pablo Bay, Suisun Bay, and Humboldt Bay (Figure 1a). We obtained environmental, urbanization, and habitat data from the California Eco‐Atlas (California Wetlands Monitoring Workgroup: https://www.ecoatlas.org). From the Eco‐Atlas, we generated landscape profiles for the HUC10 hydrologic regions surrounding each of the five focal estuaries. From each landscape profile, we extracted U.S. Census Bureau data on current human population size, human population density, and housing density (units per square mile) as proxies for the impacts of urbanization on morphological change. We also estimated the percentage of human population change for each region from the 1890s to the 2010s. We used CALVEG habitat data to estimate the percentage of land area covered by saline emergent wetland (tidal marsh), cropland, urban, and pasture habitats. The percent tidal marsh habitat reflects the contemporary amount of Savannah Sparrow habitat available in each estuary, while percentage of cropland, urban, and pasture represent the amount of landscape in the region that has been converted to human habitat. We also collected data on the number of total acres of tidal marsh habitat and the percent change in tidal marsh habitat over the last ~100 years from a variety of resources (Gerdes et al., 1974; Barnhart et al., 1992; San Francisco Estuary Institute‐Aquatic Science Center: https://www.sfei.org).

### 2.2 Data analyses

#### 2.2.1 Spatial and temporal variation in bill morphology

We first assessed the relative influence of different environmental variables on variation in bill surface area across populations of the Savannah Sparrow in California. We focused on four abiotic variables: maximum temperature (Tmax), maximum vapor pressure deficit (VPDmax), annual precipitation (Prec), and mean salinity. Vapor pressure deficit relates to the difference between observed and expected humidity given the dew point temperature and is thus influenced by both humidity and temperature as air can become more saturated with water at higher temperatures. Higher values of VPDmax are associated with lower than expected humidity with hot and dry regions, such as deserts in the southwestern United States, characterized by high levels of VPDmax (Ficklin & Novick, 2017). Evaporative water loss is less efficient in hotter and more humid environments (Gerson et al., 2014). As a consequence, selection may favor larger bills in environments with lower VPD_max_. Alternatively, the hot, dry conditions associated with higher VPD_max_ values could select for larger bills to help reduce water lost via evaporative cooling mechanisms. However, few empirical studies have explored these predictions. Minimum temperature (*T*_min_) has also been shown to influence patterns of bill morphology (Symonds & Tattersall, 2010), but we excluded it from our analyses as the subspecies *nevadensis* and *brooksi*, both of which are migratory, do not directly experience the winter minimum temperatures recorded at their breeding localities. Climate variation in the wintering grounds could also influence bill morphology variation in these two migratory subspecies. However, it is unknown where California individuals of these two subspecies winter, given that both subspecies occupy large wintering ranges. For example, *nevadensis* winters across the entire southern United States and much of northern Mexico (Pyle, 1997). Consequently, we are unable to quantify climatic conditions for wintering populations of the *nevadensis* and *brooksi* individuals in this study and our results should only be interpreted as the potential influence of breeding season climate on bill morphology. We performed a series of linear mixed‐effects models using the lmer function from the R‐package lme4 (Bates et al., 2015). For each model, surface area was the response variable, and the four environmental variables—independently and in various combinations—were fixed effects. We also included a model with the USDA Ecoregion as a fixed effect to assess whether habitat associations are a plausible explanation for bill size variation throughout California. For all models, we included tarsus length as a proxy for body size (Freeman & Jackson, 1990) and sex as fixed effects; we also generated a separate model with only tarsus length and sex as fixed effects. Finally, we incorporated the decade in which the bird sampling took place and their subspecies identity as random effects, to control for any additional variation due to these two variables alone. In total, we fit 17 linear‐fixed effects models to the data (see Table 2 for all models compared) and used a sample size‐corrected Akaike information criterion (AICc) approach to compare model fit in the AICcmodavg package in R (Mazerolle, 2019). The analyses were replicated for bill length, width, and depth.

**Table 2:** AICc results comparing the likelihood of linear mixed‐effects models

Models	K	LL	AICc	∆AICc	AICcWt
Prec:VPD_max_:*T*_max_:Salinity	21	−849.564	1,742.292	0	0.706
*T*_max_:Salinity	9	−863.354	1,744.931	2.639	0.189
*T*_max_:VPD_max_:Salinity	13	−860.718	1,747.889	5.597	0.043
VPD_max_:Salinity	9	−864.936	1,748.095	5.803	0.039
*T*_max_:Prec:Salinity	13	−861.83	1,750.114	7.822	0.014
Prec:Salinity	9	−866.594	1,751.411	9.119	0.007
Prec:VPD_max_:Salinity	13	−863.952	1,754.358	12.065	0.002
Salinity	7	−907.788	1,829.707	87.415	0
*T*_max_	7	−909.046	1,832.222	89.929	0
VPD_max_	7	−909.576	1,833.283	90.99	0
*T*_max_:Prec	9	−908.816	1,835.843	93.551	0
*T*_max_:VPD_max_	9	−908.917	1,836.045	93.752	0
Prec	7	−911.022	1,836.174	93.882	0
VPD_max_:Prec	9	−909.461	1,837.132	94.84	0
Prec:VPD_max_:*T*_max_	13	−906.465	1,839.357	97.065	0
Ecoregion	19	−936.703	1,912.264	169.971	0
Base	6	−953.599	1,919.291	176.998	0

Note

The models explore how variation in bill morphology was influenced by several fixed effects and their interactions, including annual precipitation (prec), maximum temperature (*T*_max_), maximum vapor pressure deficit (VPD_max_), and salinity. Subspecies and sampling time (decade) were included as random effects in all models. All models also include the base variables sex and tarsus (proxy for body size) as fixed effects.

We next explored the degree to which bill surface area and other bill characters vary temporally within California. To account for the effect of body size, we performed all downstream analyses on the residuals from linear regressions between tarsus length and each bill character. We first performed simple linear regressions with sampling year as an independent variable and bill surface area as the dependent variable for each of the four subspecies separately. We repeated this procedure for each of the three other bill characters.

The subspecies *alaudinus* was the temporally and spatially most sampled taxon in the dataset. Within this taxon, we went on to explore whether temporal trends in bill morphology varied across sampling localities. We divided the birds into modern (post‐1980) and historic (pre‐1980) groups. Although the choice of 1980 as the threshold was somewhat arbitrary, it was based on a large sampling gap from the late 1960s to the mid‐to‐late 1980s when few specimens were collected (see Figure S1) and corresponds to an observed shift in the rate of warming for California during the late 1970s (Cordero et al., 2011). We performed preliminary analyses to confirm that our conclusions would be robust to shifting the cutoff date between 1970 and 1995. Only when the cutoff was shifted to as recently as 2000 did results change (Table S1). We performed a series of ANCOVA analyses to assess the influence of time period (historic versus. modern), latitude, and time‐by‐latitude interactions on bill surface area, bill length, bill width, and bill depth in *alaudinus*. All statistical analyses were performed in R version 3.5.1 (https://www.r‐project.org).

#### 2.2.2 The role of genetic drift in shaping bill size change through time

We next explored whether genetic drift could explain temporal trends in bill morphology. To model the amount of trait change expected under drift, we used the following equation (Lande, 1976):

(2) $$Z=\frac{h^{2}\times \sigma^{2}\times \tau }{\mathrm{Ne}}$$

where *Z* represents the expected amount of change in a trait, *h^2^* is narrow sense heritability, σ^2^ is trait variance, τ is the number of generations over which the trait has evolved between sampling periods, and Ne is effective population size. Narrow sense heritability for bill length, width, and depth has been estimated for eastern North America populations of the Savannah Sparrow and ranges from 0.1 to 0.55 (Cava et al., 2019; Wheelwright et al., 2014). Estimates for Ne vary from 30,000 to 300,000 (Benham & Cheviron, 2020). We assumed a one‐year generation period (Wheelwright & Rising, 2008), with 120 generations of Savannah Sparrows having occurred between 1895 and 2015. Although breeding populations include individuals of various ages, the assumption of 120 generations likely overestimates the amount of bill size change that could occur due to drift. Conclusions that observed changes in bill size exceed expectations under drift would likely be conservative. Given the uncertainty around heritability and effective population size estimates, we ran 10,000 simulations in each of which we randomly drew a value from a uniform distribution bounded by upper and lower estimates for Ne and *h^2^* and used this value in equation 2 to estimate *Z*. We calculated the maximum value of *Z* (Z_max_) from across the 10,000 simulations and used the result as a threshold to test whether observed change in mean bill size exceeded the amount of bill size change expected under drift. To estimate the amount of change in bill surface area, we first z‐transformed the data so that bill surface area had a mean of 0 and variance of 1. We then performed *t* tests for bill surface area between the historic and modern birds from five separate tidal marsh populations. From these *t* test results, we extracted the difference in means between historic and modern samples as well as 95% confidence intervals around the difference in means.

#### 2.2.3 How does habitat, climate, urbanization, and migration influence change in bill morphology?

Within *alaudinus*, we next assessed whether temporal change in climate, urbanization, habitat change, or population divergence with other Savannah Sparrow populations influenced patterns of temporal change in bill morphology. For five tidal marsh populations, we calculated effect size (Cohen's *D*) for each of the four bill characters based on *t* tests between the historic (pre‐1980) and modern (post‐1980) samples. We took a similar approach to estimate effect size change in *T*_max_, *T*_min_, mean dew point temperature, VPD_max_, VPD_min_, and Prec for each site. We do not have historical data on salinity from all five of the estuaries. Salinity has been documented to increase in some California estuaries (Peterson et al., 1995), but relative differences in salinity among estuaries were likely similar historically (Marshall, 1948). While time series of salinity data would be valuable to include, increasing temperatures in higher salinity marshes may still be expected to exacerbate osmoregulatory pressures more than increasing temperatures in lower salinity marshes. Thus, despite our limitation to contemporary salinity data, we think that it is still valuable to capture the relative differences in salinity among marshes as a potential driver of bill morphology change. We divided the different variables associated with environmental change, habitat, and urbanization (see above for details on data sources) into three sets: (a) abiotic variables, including temporal change in climate parameters, and mean salinity; (b) habitat variables, such as the percentage pasture, urban, and tidal marsh habitat; and (c) parameters related to urbanization, including human population size, housing density, and percent change in human population size. For each of the three datasets, we performed a principal components analysis and saved the first two principal components from each of the three separate PC analyses (environment PC1, PC2; habitat PC1, PC2; and human PC1, PC2). To account for the influence of variation in genetic divergence (e.g., due to gene flow or divergence time) from other California Savannah Sparrow populations, we compared the influence of F*_st_* between each of the five *alaudinus* populations and *beldingi* (F*_st_ beldingi*) as well as F*_st_* between *nevadensis/brooksi* and the five *alaudinus* populations (F_st_ interior) on change in bill surface area. F*_st_* data came from a previously analyzed RADseq dataset (Benham & Cheviron, 2020). We performed a series of linear regressions with the effect size of bill change as a dependent variable and either a principal component, *F*_st_, or an individual ecological variable as the independent variable. We did not perform regression analyses with multiple variables or interactions among variables given our limited statistical power. We compared the relative fit of the various principal components, *F*_st_, and ecological variables on variation in temporal change in bill size within an AICc framework, as described above.

#### 2.2.4 Connecting bill morphology change to potential functional performance

Finally, we had hypothesized that increased bill surface area is an important adaptation to freshwater‐limited environments because it allows for increased heat dissipation, which in turn allows for reduced reliance on evaporative cooling for thermoregulation (Greenberg, et al., 2012). To explore whether temporal changes in bill morphology contributed to any water savings, we examined the following: (a) if there was any relationship between bill surface area and evaporative water loss, and, if so, (b) how much the observed changes in bill surface area contributed to increased water savings over time. To address these questions, we used previously published data on total evaporative water loss (m^−1^ g^−1^ h^−1^; TEWL) collected from some of the same individual Savannah Sparrows included in this study (Benham & Cheviron, 2020). We performed a simple linear regression with bill surface area as an independent variable and TEWL as a dependent variable. We then used results from this regression analysis to predict the influence of observed bill surface area change at the five focal estuaries on total evaporative water loss over time using the predict function in R.

## 3 RESULTS

### 3.1 Environment and spatial variation in bill morphology

**Figure 2:**
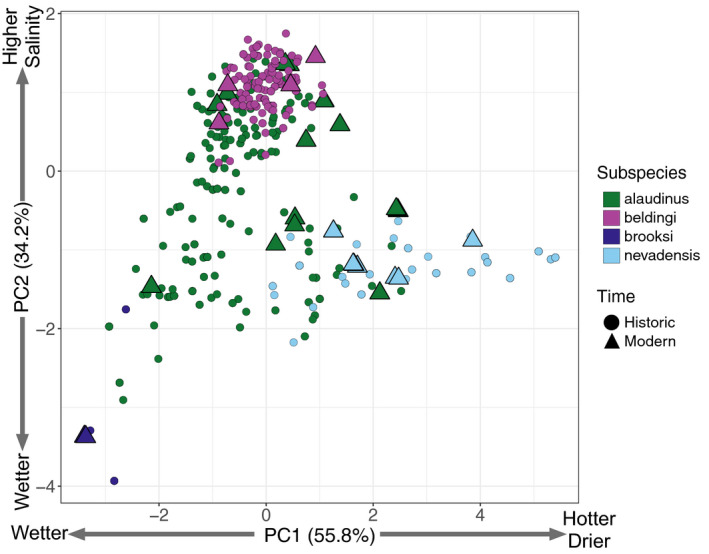
PC1 and PC2 of climate variables showing environmental differences among the four subspecies of Savannah Sparrow in California. See Table S2 for loadings

We performed principal components analysis on mean salinity, Tmax, Prec, and VPDmax data to explore patterns of climatic variation among all sampling localities in California. The first principal component explained 55.8% of the variance in the data, with the highest positive loadings associated with Tmax and VPDmax and more negative loadings associated with precipitation (Table S2). The second principal component explained 34.2% of the variation in the data, with mean salinity representing the highest positive loading and precipitation the most negative loading. Plotting PC1 versus PC2 showed that PC1 primarily separated *nevadensis* from all other subspecies, reflecting the hotter and drier habitats occupied by this subspecies during the breeding season. The other subspecies were primarily separated along PC2, with *beldingi* populations associated with more positive values linked to higher salinity and somewhat drier environments. *Brooksi* populations comprised the most negative values on PC2 and PC1, reflecting the generally high annual precipitation and freshwater habitats occupied by this subspecies. Finally, *alaudinus* populations spanned the broadest climatic space and overlapped to some extent with all other subspecies (Figure 2).

The best‐fit model explaining spatial variation in bill morphology included all four climate variables and interactions among all four variables (log‐likelihood: −849.6; AICc: 1742.3; AICc weight: 0.71; Table 2). The next best‐fit model included Tmax, salinity, and the interaction between Tmax and salinity (ΔAICc: 2.64). Within the best‐fit model, the following sets of variables were found to have significant positive effects on bill size variation: VPDmax (estimate: 0.57, *SE*: 0.21); sex (estimate: 0.27, *SE*: 0.05); tarsus length (estimate 0.11, *SE*: 0.03); interaction between *T*_max_ and Salinity (estimate: 0.27, *SE*: 0.14); interactions among precipitation, VPD_max_, and salinity variables (estimate: 0.42, *SE*: 0.18); and interactions among all four variables (estimate: 0.17, *SE*: 0.09). The interaction between precipitation, Tmax, and salinity had a significantly negative effect on bill surface area (estimate: −0.34, *SE*: 0.15; Table 3). Similarly, a linear mixed‐effects model with interactions among all four climate variables was the model that best explained variation in bill length (log‐likelihood: −954.82; AICc: 1952.78; AICc weight: 0.62) and bill width (log‐likelihood: −982.10; AICc: 2007.31; AICc weight: 0.97). For bill depth, a model including Tmax, salinity, and their interaction emerged as the best fit (log‐likelihood: −1009.52; AICc: 2037.26; AICc weight: 0.63). See Table S3 for the top five models for these other bill characters. In summary, the results suggest that no single environmental parameter fully explains variation in bill morphology, but that bills get larger in hotter, drier, and higher salinity environments in California.

**Table 3:** Results for the best‐fit linear mixed model (Bill surface area ~ precipitation*VPD_max_*T_max_*Salinity + (1|subspecies) + (1|decade)). Variables highlighted in bold were significant based on Wald chi‐square tests

Fixed Effects	β coefficient	*p*‐value	Std.Error	*t*‐value
(Intercept)	−0.23		0.41	−0.56
Prec	−0.01	.57	0.07	−0.1
**VPD_max_**	**0.57**	**.04**	**0.21**	**2.70**
*T*_max_	−0.45	.10	0.16	−2.78
Salinity	0.03	.52	0.06	0.45
**Sex**	**0.27**	**2.9e−7**	**0.05**	**5.13**
**Tarsus**	**0.11**	**4.6e−5**	**0.03**	**4.08**
Prec*VPD_max_	0.26	.54	0.22	1.18
Prec*T_max_	−0.12	.46	0.17	−0.69
VPDmax*T_max_	−0.01	.55	0.11	0.11
Prec*Salinity	0.01	.31	0.05	0.25
VPD_max_*Salinity	−0.17	.15	0.19	−0.92
*T*_max_*Salinity	**0.27**	**.03**	**0.14**	**1.91**
Prec*VPD_max_*T_max_	0.22	.81	0.12	1.91
Prec*VPD_max_*Salinity	**0.42**	**8.7e−4**	**0.18**	**2.30**
Prec*T_max_*Salinity	**−0.34**	**1.6e−4**	**0.15**	**−2.33**
VPD_max_*T_max_*Salinity	0.03	.86	0.08	0.38
Prec*VPD_max_*T_max_*Salinity	**0.17**	**.05**	**0.09**	**1.91**

### 3.2 Temporal variation in bill size

**Figure 3:**
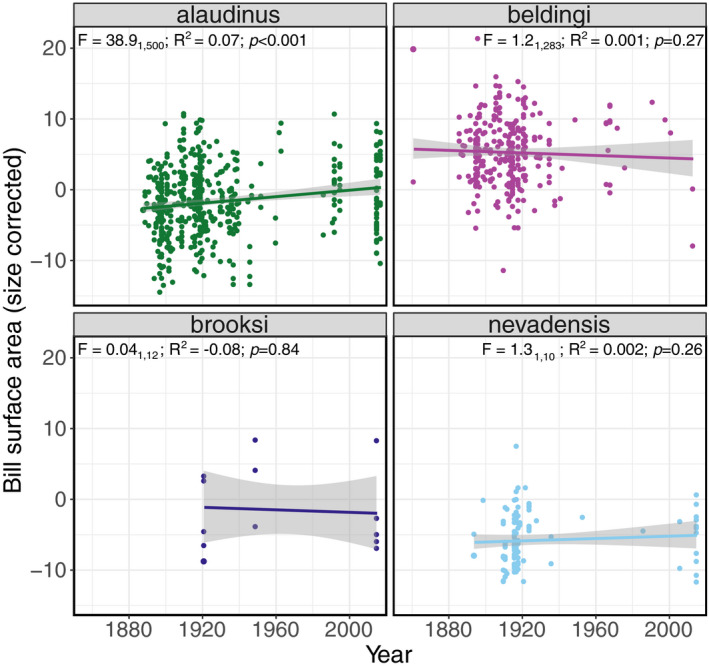
Temporal variation in bill surface area among subspecies. Only *P. s. alaudinus* (green) shows a significant temporal trend in bill surface area. Results of regression analyses that evaluate the influence of year on variation in bill surface area are reported on upper portion of each plot

Only *alaudinus* showed a significant, but subtle increase in bill surface area over time in California (adjusted *R* ^2^: 0.07; *p*‐value < .001; Figure 3). *Alaudinus* exhibited significant increases in other bill characters: bill length (adjusted *R* ^2^: 0.027; *p* < .001), bill width (adjusted *R* ^2^: 0.021; *p* < .001), and bill depth (adjusted *R* ^2^: 0.038; *p* < .001). *Beldingi* also showed a significant increase in bill depth over time (adjusted *R* ^2^: 0.0014; *p* < .05; Fig. S2; Table S4).

**Figure 4:**
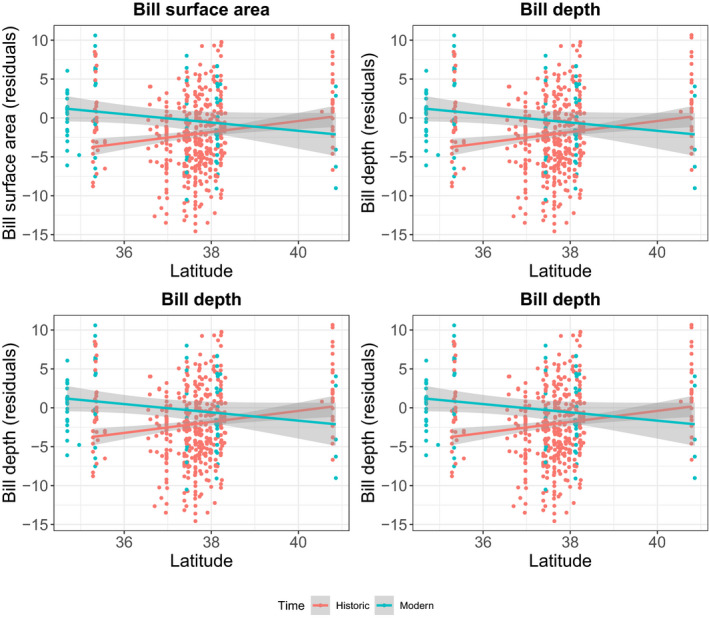
Temporal differences in latitudinal trends for different bill characters within *P. s. alaudinus*. Red points and trendlines represent specimens collected prior to 1980, while blue points and trendlines represent birds sampled after 1980. See Table 4 for ANCOVA results

Within *alaudinus*, all bill characters increased in the southern part of the distribution, but either did not change or decreased over time in the northern populations (Figure 4). ANCOVA analyses support a significant effect on bill surface area over time period (*F*_1,498_ = 13.34; *p* < .001) and of time period‐by‐latitude interaction (*F*_1,498_ = 11.67; *p* < .001), but not of latitude (*F*_1,498_ = 3.20; *p* > .05; Table 4). The effect of time period was significant on all other bill characters except bill depth; only latitude had a significant effect on bill length. Finally, the interaction between time period and latitude had a significant effect on all characters except bill length (Table 4).

**Table 4:** ANCOVA results examining the influence of time period (historic vs. modern), latitude, and time*latitude interactions on different bill characters within the subspecies *P. s. alaudinus*

Bill character	Time period	Latitude	Time period:Latitude interaction
Bill surface area	13.34***	3.20^ns^	11.67***
Bill length	9.42**	5.82*	1.45^ns^
Bill width	28.05***	0.35^ns^	8.07**
Bill depth	0.06^ns^	1.63^ns^	22.30***

Note

F‐statistics for each model are listed; asterisks signify *p*‐values. ns: *p* > .05; *p < .05; **p < .01; ***p < .001.

### 3.3 The influence of genetic drift on bill size change

**Figure 5:**
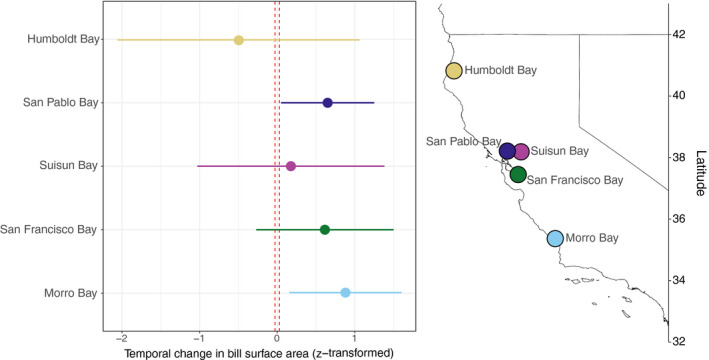
Variation in the degree of temporal change among populations of *P. s. alaudinus*. Colored dots signify difference in mean bill surface area (z‐transformed) between historic and modern specimens, and colored lines indicate the 95% confidence interval. Dashed red lines signify the amount of bill morphology change expected under drift. Different colors correspond to the five different estuaries from which we had sufficient historical and modern sampling. The geographic distribution of these estuaries is illustrated on the map of California in the right‐hand panel

Our models of bill morphology change indicated that very little change in bill morphology (−0.03 < *Z*_max_ < 0.03) would be expected over the passage of 120 generations due to genetic drift alone (Figure S3). When compared to observed bill morphology change, two populations showed significant change in bill surface across time—those from Morro Bay and San Pablo Bay—with both exhibiting a greater amount of change than expected under drift (Figure 5). Although the mean difference in bill surface area between historic and modern populations at San Francisco Bay, Suisun Bay, and Humboldt Bay exceeded the amount of change expected under drift, the 95% confidence intervals of the *t* test results encompassed *Z_max._*

### 3.4 The influence of climate change, habitat change, urbanization, and population structure on temporal variation in bill size

**Figure 6:**
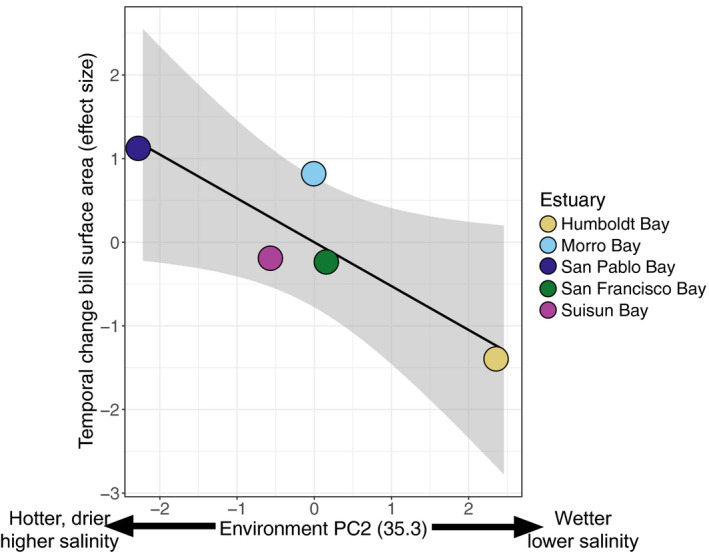
Relationship between degree of temporal change in bill surface area and temporal change in abiotic variables across five populations of *P. s. alaudinus* (plotted on map in right panel). Bill surface area is the effect size (Cohen's D) of bill change between historic and modern groups. Environment PC2 was generated from effect size change in temperature, precipitation, humidity, and salinity variables. Loadings on the second principal component showed negative values associated with climate getting hotter and drier in tidal marshes with higher salinity, while positive loadings were associated with increasing precipitation and lower salinity tidal marshes. See Table 5 for statistical results of linear regression models

We evaluated the contribution of climate change, habitat change, urbanization, and population structure on variation in the degree of temporal bill morphology change using principal components analysis (see Table S4 for loadings) and AICc approaches (see Tables S6 & S7 for AICc results). The second principal component of the environmental variables best explained variation in the magnitude of bill surface area change (Adjusted *R* ^2^: 0.702; *p* = .048; Table 5). This finding suggests that the greatest increases in bill surface area occurred in populations that occupy higher salinity tidal marshes that have become hotter and drier over time (Figure 6). Temporal variation in Tmin was the second best model, but the relationship with bill surface area change was not significant (ΔAICc: 1.14; adjusted *R* ^2^: 0.66; *p* = .06; Table S6). Temporal variation in bill length among sites was significantly explained by both environment PC2 (Adjusted *R* ^2^: 0.79; *p* = .028) and habitat PC1 (ΔAICc: 0.06; Adjusted *R* ^2^: 0.790; *p* = .028; Figure S4). Variation in F_st_ divergence between *beldingi* and each of the five populations best explained variation in bill width change among sites (Table 5). Populations with little F*_st_* divergence from *beldingi* showed the greatest bill width change (Figure S5). Variation in temporal change in bill depth, on the other hand, was best explained by variation in F*_st_* between *brooksi/nevadensis* and coastal *alaudinus* populations (Figure S5).

**Table 5:** Linear regression results assessing the influence of change in environment, habitat, or urbanization on the degree of change in each bill character among the five focal *P. s. alaudinus* populations

Models	*F*‐statistic	*df*	Adjusted *R* ^2^	*p*‐value
Surface area ~ Environment PC2	10.42	1, 3	0.702	.048
Bill length ~ Environment PC2	16.07	1, 3	0.790	.028
Bill width ~ Fst *beldingi*	12.03	1, 3	0.734	.040
Bill depth ~ Fst interior	35.32	1, 3	0.896	.009

Note

Models listed here are the best‐fit models identified using an AICc approach (see Table S5 & S6).

### 3.5 Functional significance of bill morphology change through space and time

**Figure 7:**
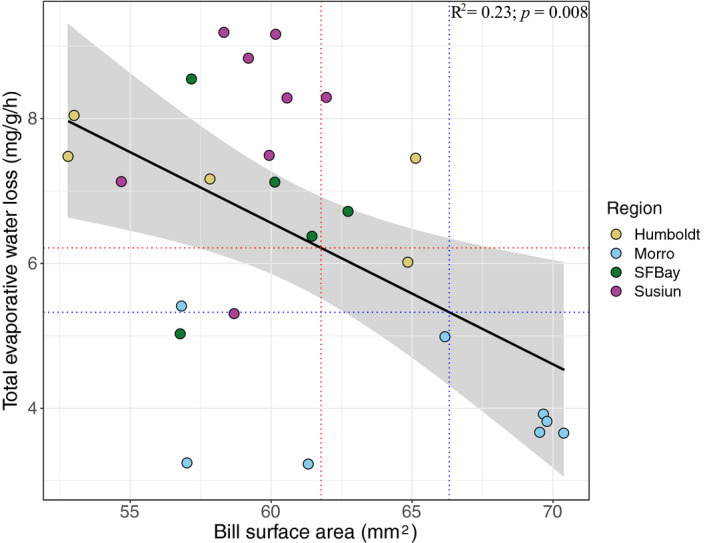
Relationship between bill surface area and total evaporative water loss (mg^−1^ g^−1^ hr^−1^). Points are colored by population (see sampling map from Figure 5). Red dotted lines represent historical bill size and estimated TEWL from the Morro Bay population, and blue dotted lines the mean modern bill surface area and TEWL in Morro Bay birds. Arrows signify the predicted direction of shift through time in bill surface area and TEWL

Within *alaudinus,* we found a significant correlation between bill surface area and total evaporative water loss (mg^−1^ g^−1^ hr^−1^; adjusted *R* ^2^: 0.23; *p* = .008; Figure 7). Assuming that this trend holds true across the temporal scales assessed here, the amount of bill surface area change across the five focal estuaries varied from an increase of 0.46 mg^−1^ g^−1^ hr^−1^ in the population at Humboldt Bay to a decrease of 0.89 mg^−1^ g^−1^ hr^−1^ at Morro Bay (Table S8).

## 4 DISCUSSION

Museum specimens offer an unparalleled resource for understanding how organisms have responded to the past century of human‐mediated ecological change. However, for many of the functional traits that can be measured on specimens, temporal variation could reflect the influence of many different ecological pressures or demographic factors. This was evident in our analyses of spatial and temporal variation of Savannah Sparrow bill morphology, in which no single environmental variable best explained variation in bill morphology. Rather, spatial variation in bill size reflected interactions among the variables of temperature, precipitation, humidity, and salinity. Similarly, the greatest temporal changes in bill surface area within the *alaudinus* subspecies were observed in populations occupying higher salinity marshes that have experienced increases in maximum temperature and decreases in humidity. Our results illustrate the power of integrating data from both spatial and temporal series of specimens in order to accurately evaluate the influence of multiple ecological pressures on temporal variation in functionally significant, morphological characters.

### 4.1 The influence of the abiotic environment on bill size variation

Our results have important implications for interpreting patterns of bill morphology variation in association with climate. The importance of the avian bill as a thermoregulatory organ suggests that temperature may play an important role in shaping spatial patterns of bill variation (Tattersall et al., 2017). Temperature variation has been found to explain both inter‐ and intra‐specific variation in bill size across a diverse set of taxa (Campbell‐Tennant et al., 2015; Danner & Greenberg, 2015; Symonds & Tattersall, 2010). However, in most of these studies, temperature was the only climatic variable explored, and several species (46 of 110 in one study) failed to exhibit patterns consistent with this hypothesis (Tattersall et al., 2017). Similarly, the evidence of a relationship between temporal variation in bill size and temperature is mixed (Campbell‐Tennant et al., 2015; Miller et al., 2018). Our modeling has found that Tmax significantly explains spatial variation in bill size only when interacting with salinity, precipitation, and VPDmax (Table 3). Together, these patterns suggest that the influence of temperature on bill size variation will in many cases be mediated through other climatic and environmental parameters that need to be considered.

Humidity is one variable that may interact with temperature in important ways to shape bill size variation. In theory, selection for larger bills may be greatest in hotter and more humid climates, as evaporative cooling is highly inefficient at high humidity (Gerson et al., 2014; van de Ven et al., 2016). Gardner et al. (2016) found larger bill surface areas in Australian honeyeater (Melliphagoidea) populations experiencing higher humidity and cooler summers. In contrast, Northern Cardinals (*Cardinalis cardinalis*) exhibit a negative relationship between bill size and humidity in populations experiencing lower winter temperatures, but no relationship between these two variables in warmer climates (Miller et al., 2018). Our best‐fit model also indicates a positive association between bill surface area and VPDmax (Table 3), where higher VPD_max_ signifies lower humidity than expected. While the best‐fit model involved interactions between VPD_max_ and other climatic variables, our results are more consistent with patterns reported for Northern Cardinals than for either honeyeaters or theoretical predictions.

Evidence for increased bill size under drier conditions is instead consistent with a body of work, suggesting that selection in freshwater‐limited environments should favor reductions in evaporative water loss to conserve water and that increased heat dissipation capacity across a larger bill surface area facilitates a reduction in evaporative water loss (Greenberg, et al., 2012). This relationship is thought to contribute to the frequently observed increase in bill size in high salinity environments (Grenier & Greenberg, 2005; Luther & Greenberg, 2011) and to the significant correlation between bill size variation and maximum summer temperatures found across tidal marsh sparrows (Greenberg, et al., 2012). Additional research on Song Sparrows (*Melospiza melodia*) confirms that larger‐billed coastal birds can dissipate more heat from across the bill surface than can smaller‐billed individuals from interior localities (Greenberg, et al., 2012). We also found larger spatial and temporal increases in bill size in tidal marsh populations of the Savannah Sparrow, with greater increases being associated with higher temperatures and drier conditions. Together, patterns of bill size variation in association with humidity and salinity suggest that the evolution of larger bills does play an important thermoregulatory role in freshwater‐limited environments, but the exact influence of water availability is likely mediated through variation in temperature.

### 4.2 The functional significance of larger bills

Despite the evidence outlined above, the role of increased heat dissipation across a larger bill surface in actually facilitating reduced water loss has remained untested. In support of this prediction, we found a significantly negative relationship between total evaporative water loss and bill surface area (Figure 7). Extrapolating from this finding, we estimated that the observed increases in bill surface area over time in Savannah Sparrows at San Pablo Bay and Morro Bay could result in decreases in water loss on the magnitude of 0.60 to 0.89 mg^−1^ g^−1^ hr^−1^ (8.2%–14.3%), respectively (Table S8), corresponding to a daily savings of 10.8% and 16.2% of total expected water loss given the average body mass of individuals from these two populations (equation from Dawson, 1982). In Song Sparrows, a 13.1% increase in bill surface area corresponded to a 33% increase in the amount of heat dissipated from across the bill surface (Greenberg, et al., 2012). Given this increase in heat dissipation from across the bill surface, the authors estimated that larger‐billed, coastal Song Sparrows could theoretically reduce evaporative water losses by 9.7 mg/hr or 7.7% of daily water losses. Our data suggest that smaller increases in bill surface area over time correspond to greater increases in water savings (e.g., 7.37% increase in bill surface area and 16.2% reduction in water loss). Larger bills may also play a more direct role in reduced respiratory water loss (part of total evaporative water loss) through increased surface area of the nasal conchae. These respiratory surfaces function within a counter‐current gradient system that warms inspired and cools expired air. As the air cools during exhalation, water condenses on the nasal conchae and is then reabsorbed by the bird (Geist, 2000; Schmidt‐Nielsen et al., 1970). An association between increased nasal conchae surface area and bill size has been shown in coastal Song Sparrows (Danner et al., 2017), though the contribution of these differences to water savings remains untested.

Functional inferences from our data are limited in a number of ways. First, our analyses were limited to water loss data collected at rest within the thermal neutral zone (28°C). It is unknown whether water losses in coastal Savannah Sparrow populations will also be curtailed under thermal stress at higher temperatures where water loss increases dramatically in most birds (Bartholomew, 1972). Despite increasing temperatures at sampling localities along the California coast, maximum temperatures rarely exceed 30°C, suggesting that the available data on water loss are ecologically relevant. It is further possible that bird bills are more effective as thermal radiators at moderate temperatures. For example, Southern Ground Hornbills (*Bucorvus leadbeateri*) rely more heavily on their bills for thermal radiation at moderate temperatures (22–28°C) where there is a more significant thermal gradient between the bill and the environment (van Vuuren et al., 2020). California Song Sparrows also exhibit a trend toward larger bill size with warming climates, but decreases in their bill size in the hottest climates (Greenberg & Danner, 2012). Second, we report only a correlation between bill size and water loss. It remains to be tested whether larger bills play a direct role in facilitating reduced water loss. This correlation could also arise if spatially correlated selective pressures operate independently on bill size and evaporative water loss. Demographic constraints could also play a role, as gene flow was previously found to influence evaporative water loss across broader scales in Savannah Sparrows (Benham & Cheviron, 2020). Finally, the fitness consequences of the levels of water loss reduction we report here remain unclear. Water loss varies inversely with body size, with daily water loss becoming a significant proportion of body weight (e.g., 15%–25%) among small‐bodied bird species like sparrows (Bartholomew & Cade, 1963); even the modest reductions in water loss we report could mitigate the greater water loss experienced in small birds. Although the levels of water loss reported for small birds were previously thought to exceed metabolic water production (Bartholomew, 1972), phylogenetically controlled analyses suggest that small birds may gain some water from metabolism (Williams, 1996). With reduced water loss, sparrows could gain more of their daily water needs from metabolic water production. Such water savings could also provide fitness benefits for smaller‐billed birds by allowing them to forage and sing for longer periods under hot conditions (Luther & Danner, 2016). Addressing these different considerations will ultimately require experiments that directly link patterns of bill size variation, heat dissipation, and evaporative water loss together with an evaluation of the fitness consequences of this variation.

### 4.3 Diet and bill morphology

Although extensive evidence suggests that foraging ecology significantly shapes intra‐specific variation in bird bills (Benkman, 2003; Boag & Grant, 1981; Bosse et al., 2017), our models do not explicitly account for variation in diet. However, it is unlikely that dietary changes explain all of the patterns we observed. Diets might be expected to differ most between distinct habitats, yet we found that models including ecoregion were among the poorest fitting models to observed spatial variation in bill size (Table 2). Likewise, we found patterns of temporal change–with the exception of bill length—to be best explained by climate change and salinity. Even for bill length, the least amount of change through time was observed in Humboldt Bay, an area in which Savannah Sparrows expanded their range into cattle pasture as surrounding marshlands were diked and drained for pasture (Fitton, 2008). In contrast, all bill dimensions increased the most in Morro Bay, where tidal marsh habitat has expanded in recent decades (Gerdes et al., 1974). It is also possible that observed correlations between climate change and bill morphology reflect the influence of climate change on temporal variation in food resources, which in turn drives changes in bill size. Our work focuses primarily on metrics of bill surface area and size. An additional focus on aspects of bill shape could reveal different associations with habitat change or diet (Friedman et al., 2019). To resolve these issues, future work could investigate dietary shifts using isotope data from feathers (McMahon et al., 2019; Norris et al., 2007; Walsh et al., 2016) or data on patterns of changing seed size from herbaria. These data would allow for simultaneous evaluation of the influence of diet and climate on different dimensions of bill morphology. One of the few studies that simultaneously accounts for both climate and dietary effects on bill size found that both temperature variation and foraging ecology significantly explain spatial variation in bill size and shape across Australian honeyeaters (Friedman et al., 2019). This evidence suggests that selection likely operates on bird bills to optimize them for multiple functions.

### 4.4 Demography and morphological change

Population demography, such as variation in effective population size or patterns of gene flow, could also influence spatial and temporal patterns of morphological variation (Lenormand, 2002; Wright, 1932). Few studies of museum specimens attempt to account for the influence of drift on temporal change (but see Assis et al., 2016). Our modeling suggests that genetic drift explains few temporal changes in bill size (Figure S4); however, we could completely exclude drift in only two of the five populations we examined (Figure 5). In the other three populations, the lack of temporal variation could be due to drift or to the lack of strong selection pressures driving temporal change. The latter explanation seems more likely, given that the lack of change correlates with patterns of environmental change through time (Figure 6) and that previous studies have found little difference in effective population size among these populations (Benham & Cheviron, 2020). In line with expectations from population genetics theory (Wright, 1932), our results suggest that—at least in populations with large effective population sizes—drift is unlikely to play a significant role in shaping temporal change over short time scales.

Anthropogenic change could also contribute to shifting patterns of gene flow among populations (Grabenstein & Taylor, 2018), but to our knowledge, the influence of gene flow patterns on temporal change in museum specimens has not been evaluated. We found that greater *F*_st_ divergence from *beldingi* was negatively correlated with bill width (Figure S5). While lower *F*_st_ divergence can reflect either recent divergence or greater gene flow, this pattern is consistent with greater gene flow between the wider‐billed *beldingi* subspecies and southern populations of *alaudinus*. We found an opposite pattern for bill depth among shallower‐billed, interior populations of the Savannah Sparrow: For them, *F*_st_ divergence was positively correlated with bill depth. This finding is consistent with gene flow from interior birds leading to shallower‐billed *alaudinus* populations in the north (e.g., Humboldt Bay) where *alaudinus* comes into close proximity with the upland subspecies *brooksi*. These patterns suggest that gene flow may constrain divergence in bill depth and width between populations, as has been shown for a number of traits and species (Nosil & Crespi, 2004; Postma & Noordwijk, 2005; Stuart et al., 2017) including bill length in hummingbirds (Benham & Witt, 2016). Gene flow could additionally provide critical genetic variation for selection to act upon (Taylor & Larson, 2019). This may be especially true in Morro Bay, which is known to be an admixture zone between the *beldingi* and *alaudinus* subspecies (Benham & Cheviron, 2019), but more work is needed to determine whether gene flow has contributed to adaptive change in bill size within this population.

Genetic drift and gene flow can only influence patterns of bill size variation if this trait has a genetic basis. Bill size and shape have been shown to be heritable in several bird species (Boag & Grant, 1978), including other New World Sparrows (Ballentine & Greenberg 2010; Smith & Zach, 1978). Genomic analyses have also identified a number of genes associated with variation in bill morphology (Abzhanov et al., 2006; Bosse et al., 2017; Lamichhaney et al., 2015; Mallarino et al., 2012; vonHoldt et al., 2018). In addition to drift and gene flow, variation in pools of standing genetic variation among sites could also shape observed heterogeneity in temporal bill size variation (Barrett & Schluter, 2008). For example, if certain populations contained more genetic variation in genes that underpin the formation of the bill, selection on different alleles could lead to greater morphological change in those populations. Differential selection on allelic variation among different bill genes across the species range could also contribute to observed patterns of morphological variation. Genomic sequencing of modern and historic samples would enable the tracking of changes in functional alleles through time and thereby provide valuable insights into the genetic basis of bill morphology and functional genomic responses to climate change (Bi et al., 2019). Finally, we cannot completely exclude the possibility that plasticity could also contribute to some of the observed patterns. Birds are known to exhibit seasonal variation in growth of the keratinized sheath surrounding the bone of bird bills (Greenberg et al., 2013). Developmental environment has also been linked to variation in bill morphology (James, 1983, 1991). Although plasticity could contribute to some of the observed bill morphology patterns, plastic increases in bill size in response to climate would still have important functional consequences (Figure 7) that facilitate acclimation to anthropogenic climate change.

### 4.5 Drivers of heterogeneous temporal change in morphological characters

Beyond bill morphology, a wide range of morphological traits, physiological traits, phenology, and distributions may all be expected to exhibit similarly complex patterns of temporal change in response to anthropogenic change. This complexity could be due to temporal variation in human‐mediated ecological pressures being nonuniform in space. For example, in response to asynchronous patterns of climate change, one population of arctic breeding Hudsonian Godwits (*Limosa haemastica*) has advanced its arrival date to the breeding grounds by nine days while a second population has delayed its arrival by more than 10 days (Senner, 2012). Spatial variation in precipitation and temperature change over time has also contributed to heterogeneous range shifts in birds (Tingley et al., 2012) and in mammals (Rowe et al., 2015) in the Sierra Nevada Mountains of California. Many traits that can be measured on museum specimens, like bill size, also play important roles in multiple different organismal functions. The selective pressures imposed by different interacting anthropogenic pressures may further contribute to idiosyncratic responses. For example, variation in cranial morphology in mammals is associated with foraging, sensory, physiological, and locomotive functions (Holmes, Hammond, et al., 2016, 2016; Walsh et al., 2016). Similar to the spatially heterogeneous patterns of temporal change we found for bill size in Californian populations of Savannah Sparrow, spatially variable patterns of temporal change in cranial shape among Deer Mice (*Peromyscus maniculatus*) have been associated in part with different histories of climate change in the three sampling transects across the Sierra Nevada Mountains of California (Holmes, Hammond, et al., 2016, 2016). Together with our results, the examples above suggest that analyses of museum specimens that examine spatially heterogeneous patterns of temporal change in ecological pressures and traits will ultimately improve scientific understanding of specific drivers of observed population responses. This work will, in turn, contribute to more nuanced predictions about the capacity for populations to respond adaptively to anthropogenic change in the future.

## 5 CONCLUSIONS

We have brought together a series of analyses that investigated: (a) the influence of environmental parameters on spatial variation in bill morphology; (b) the influence of anthropogenic change on patterns of temporal change in bill morphology; and (c) the relationship between bill morphology variation and evaporative water loss. Integrating these analyses we found that, both spatially and temporally, bill surface area increased in Savannah Sparrow populations occupying higher salinity tidal marshes that have gotten hotter and drier over time. This variation in bill morphology was consistent with our predictions that selection favors larger bills in hotter, freshwater‐limited environments to increase heat dissipation and reduce evaporative water loss. We did not find any evidence for bill morphology change in response to habitat change or urbanization. These results suggest that bill morphology change is an important adaptive, but not universal, response to future climate change. This work underscores the importance of taking into account a broad range of selective pressures and demographic parameters that may be shaping patterns of temporal change in morphological characters. Our approach can be replicated across a broad range of taxa and facilitate the extraction of greater insights into population responses to anthropogenic change from the millions of specimens housed in natural history collections around the globe.

## Supporting information

Supplementary Material

## Data Availability

All morphological and environmental data along with code for performing analysis are available on the Dryad Data Archive (https://doi.org/10.6078/D1CT4G).
