# Supplementary material for: The influence of spatially heterogeneous anthropogenic change on bill size evolution in a coastal songbird
Source: Evol Appl. 2020 Oct 21;14(2):607–24. doi: 10.1111/eva.13144 (PMC7896719; doi:10.1111/eva.13144)
Supplement: Supplementary file 1 — Supplementary Material [file EVA-14-607-s001.docx]

Evolutionary Applications

*Supplementary Material for:*

**The influence of spatially heterogenous anthropogenic change on bill size evolution in a coastal songbird**

SUPPLEMENTAL FIGURES:

**Supplemental figure S1:** Spatial and temporal distribution of specimens measured for each subspecies. Sample sizes represent all samples available for analysis prior to filtering for downstream analyses.

**Supplemental Figure S2:** Variation in bill characters among subspecies and across time. Only *P. s. alaudinus* shows a significant temporal trend in bill length and bill width. Both *P. s. alaudinus* and *P. s. beldingi* exhibit significant temporal trends in bill depth. See supplemental Table S3 for results from linear regressions.

**Supplemental Figure S3:** Expected amount of bill size change (*Z*) through genetic drift over 120 generations. Distribution of *Z* based on 10,000 simulations while varying estimates for effective population size from 30,000 to 300,000 and heritability (*h^2^*) from 0.1 to 0.55.

**Supplemental figure S4:** Plots showing the relationship between the degree of temporal bill length change across five different populations of *P. s. alaudinus* in coastal California and the two best-fit models (see supplemental Table S6). (Left panel) The influence of the second principal component of environmental variables on bill length change. (Right panel) The influence of the first principal component of habitat variables on bill length change. Bill length change is presented as effect sizes (Cohen's D) calculated from t-tests between historic and modern specimens.

**Supplemental figure S5:** (left panel) Plot showing the relationship between the degree of temporal bill width change across five different populations of *P. s. alaudinus* in coastal California and Fst divergence between the *P.s. beldingi* subspecies and each of the five *P. s. alaudinus* subspecies. (right panel) Plot showing the relationship between the degree of bill depth change across five different populations of *P. s. alaudinus* in coastal California and Fst divergence between the *P.s. brooksi/nevadensis* subspecies (interior) and each of the five *P. s. alaudinus* subspecies. Bill width and depth change is presented as effect sizes (Cohen's D) calculated from t-tests between historic and modern specimens.

SUPPLEMENTAL TABLES:

**Supplemental Table S1:** Comparison of ANCOVA results using different cutoff thresholds for time period that examine the influence of time period (historic vs. modern), latitude, and time*latitude interactions on different bill characters within the subspecies *P. s. alaudinus* (see Table 4). Different cutoffs are 1970, 1980, 1990, 1995, and 2000*.* Samples sizes between historic (n-historic) and modern (n-modern) samples are listed in header. F-statistics for each model are listed; asterisks signify p-values. Cells of table highlighted in yellow denote results where significance and conclusions differ from the cutoff of 1980 used in the main paper.

| **Modern vs. historic cutoff 1970 (n-historic = 444; n-modern = 82)** | | | |
| --- | --- | --- | --- |
| **Bill character** | **Time period** | **Latitude** | **Time period:Latitude interaction** |
| Bill surface area | 13.34*** | 3.20^ns^ | 11.67*** |
| Bill length | 9.42** | 5.82* | 1.45^ns^ |
| Bill width | 28.05*** | 0.35^ns^ | 8.07** |
| Bill depth | 0.06^ns^ | 1.63^ns^ | 22.30*** |
|  |  |  |  |
| **Modern vs. historic cutoff 1980 (n-historic = 444; n-modern = 82)** | | | |
| **Bill character** | **Time period** | **Latitude** | **Time period:Latitude interaction** |
| Bill surface area | 13.34*** | 3.20^ns^ | 11.67*** |
| Bill length | 9.42** | 5.82* | 1.45^ns^ |
| Bill width | 28.05*** | 0.35^ns^ | 8.07** |
| Bill depth | 0.06^ns^ | 1.63^ns^ | 22.30*** |
|  |  |  |  |
| **Modern vs. historic cutoff 1990 (n-historic = 448; n-modern = 78)** | | | |
| **Bill character** | **Time period** | **Latitude** | **Time period:Latitude interaction** |
| Bill surface area | 14.48*** | 3.13^ns^ | 12.61*** |
| Bill length | 10.03** | 5.66* | 1.61^ns^ |
| Bill width | 28.84*** | 0.44^ns^ | 8.62** |
| Bill depth | 0.17n^s^ | 1.74^ns^ | 23.93*** |
|  |  |  |  |
| **Modern vs. historic cutoff 1995 (n-historic = 467; n-modern = 59)** | | | |
| **Bill character** | **Time period** | **Latitude** | **Time period:Latitude interaction** |
| Bill surface area | 8.45** | 0.75^ns^ | 7.92** |
| Bill length | 10.0** | 2.73ns | 1.55ns |
| Bill width | 20.66*** | 3.12ns | 9.11** |
| Bill depth | 1.48ns | 1.13ns | 8.24** |
|  |  |  |  |
| **Modern vs. historic cutoff 2000 (n-historic = 479; n-modern = 47)** | | | |
| **Bill character** | **Time period** | **Latitude** | **Time period:Latitude interaction** |
| Bill surface area | 5.51* | 031^ns^ | 6.04* |
| Bill length | 12.08** | 1.62ns | 4.67* |
| Bill width | 15.64*** | 5.12* | 11.88*** |
| Bill depth | 7.67** | 1.64ns | 0.008ns |

***p<0.001; **p<0.01; *p<0.05

**Supplemental Table S2:** Loadings from principal components analysis on four environmental variables from all California populations sampled.

| **Parameter** | **PC1** | **PC2** | **PC3** | **PC4** |
| --- | --- | --- | --- | --- |
| Mean salinity | -0.126 | 0.779 | 0.581 | -0.198 |
| Max. temperature | 0.642 | -0.011 | 0.382 | 0.664 |
| Annual precipitation | -0.398 | -0.593 | 0.699 | -0.027 |
| Max. Vapor pressure deficit | 0.642 | -0.203 | 0.166 | -0.720 |
| **Standard deviation** | 1.515 | 1.852 | 0.614 | 0.187 |
| **Proportion of variance** | 0.558 | 0.342 | 0.092 | 0.008 |
| **Cumulative proportion** | 0.558 | 0.900 | 0.991 | 1.000 |

**Supplemental Table S3:** AICc results comparing the likelihood of linear mixed effects models. The models explored how variation in different bill characters was influenced by several fixed effects and their interactions, including: annual precipitation (prec), maximum temperature (Tmax), maximum vapor pressure deficit (VPDmax), and salinity. All models also include tarsus and sex as fixed effects. Subspecies and sampling time (decade) were included as random effects in all models. Tables show top 5 models only.

| **Bill length** | | | | | | |
| --- | --- | --- | --- | --- | --- | --- |
| **Model** | **K** | **AICc** | **∆AICc** | **AICcWt** | **Cum.Wt** | **LL** |
| Prec:VPDmax:Tmax:Salinity | 21 | 1952.78 | 0.00 | 0.62 | 0.62 | -954.82 |
| Tmax:Salinity | 9 | 1955.39 | 2.62 | 0.17 | 0.79 | -968.58 |
| VPDmax:Salinity | 9 | 1956.14 | 3.36 | 0.12 | 0.91 | -968.96 |
| Tmax:VPDmax:Salinity | 13 | 1958.13 | 5.35 | 0.04 | 0.95 | -965.84 |
| Prec:Salinity | 9 | 1958.94 | 6.16 | 0.03 | 0.98 | -970.35 |
|  |  |  |  |  |  |  |
| **Bill width** | | | | | | |
| **Model** | **K** | **AICc** | **∆AICc** | **AICcWt** | **Cum.Wt** | **LL** |
| Prec:VPDmax:Tmax:Salinity | 21 | 2007.31 | 0 | 0.97 | 0.97 | -982.10 |
| Tmax:Prec:Salinity | 13 | 2014.57 | 7.25 | 0.03 | 0.99 | -994.06 |
| Tmax:VPDmax:Salinity | 13 | 2017.79 | 10.48 | 0.005 | 1.00 | -995.67 |
| Prec:Salinity | 9 | 2019.96 | 12.65 | 0.002 | 1.00 | -1000.87 |
| Prec:VPDmax:Salinity | 13 | 2021.62 | 14.31 | 0.001 | 1.00 | -997.59 |
|  |  |  |  |  |  |  |
| **Bill depth** | | | | | | |
| **Model** | **K** | **AICc** | **∆AICc** | **AICcWt** | **Cum.Wt** | **LL** |
| Tmax:Salinity | 9 | 2037.26 | 0 | 0.63 | 0.63 | -1009.52 |
| Tmax:VPDmax:Salinity | 13 | 2039.19 | 1.93 | 0.24 | 0.87 | -1006.37 |
| VPDmax:Salinity | 9 | 2042.23 | 4.97 | 0.05 | 0.92 | -1012.00 |
| Prec:VPDmax:Tmax:Salinity | 21 | 2042.26 | 4.99 | 0.05 | 0.97 | -999.55 |
| Tmax:Prec:Salinity | 13 | 2044.26 | 7.00 | 0.02 | 0.99 | -1008.91 |

**Supplemental Table S4:** Influence of sampling year on variation in bill morphology for each subspecies of California Savannah Sparrow. Linear regression results presented as adjusted R^2^ (F-statistic _degrees of freedom_).

| **Subspecies** | **Bill length** | **Bill width** | **Bill depth** |
| --- | --- | --- | --- |
| *P. s. alaudinus* | **0.027*** (14.84_1,500_)** | **0.021*** (11.84_1,500_)** | **0.038*** (20.95_1,500_)** |
| *P. s. beldingi* | -0.002 (0.49_1,283_) | -0.003 (0.01_1,283_) | **0.0014* (5.10_1,283_)** |
| *P. s. brooksi* | -0.078 (0.07_1,12_) | -0.012 (0.84_1,12_) | -0.074 (0.10_1,12_) |
| *P. s. nevadensis* | -0.003 (0.60_1,104_) | -0.009 (0.026_1,104_) | -0.007 (0.258_1,104_) |

***p<0.001; **p<0.01; *p<0.05

**Supplemental Table S5:** Loadings for principal components analysis of change in different climate variables and salinity (modern measurements only), parameters associated with increasing urbanization over time, and changing habitats through time.

| **Environment PCA** | | | | | |
| --- | --- | --- | --- | --- | --- |
| **Parameter** | **PC1** | **PC2** | **PC3** | **PC4** | **PC5** |
| Tmax | 0.132 | -0.528 | 0.379 | -0.171 | 0.458 |
| Tmin | -0.024 | 0.574 | -0.149 | -0.666 | -0.008 |
| Prec | -0.348 | 0.134 | 0.686 | -0.101 | -0.098 |
| VPDmax | 0.306 | -0.456 | -0.036 | -0.493 | -0.581 |
| VPDmin | -0.480 | 0.052 | 0.220 | 0.002 | -0.110 |
| TDmean | 0.450 | 0.241 | -0.045 | 0.136 | 0.358 |
| SampleSalinity | -0.407 | -0.208 | -0.443 | 0.275 | -0.198 |
| MeanSalinity | -0.412 | -0.247 | -0.342 | -0.424 | 0.513 |
| Standard deviation | 2.029 | 1.680 | 0.979 | 0.324 | 0.000 |
| Proportion of variance | 0.514 | 0.353 | 0.120 | 0.013 | 0.000 |
| Cumulative proportion | 0.514 | 0.867 | 0.987 | 1.000 | 1.000 |
|  |  |  |  |  |  |
| **Urbanization PCA** | | | | | |
| **Parameter** | **PC1** | **PC2** | **PC3** | **PC4** |  |
| PctPopulationChange | 0.342 | 0.939 | 0.029 | 0.034 |  |
| CurrentPop | 0.544 | -0.168 | -0.810 | -0.139 |  |
| HumanPopDensity | 0.543 | -0.190 | 0.514 | -0.636 |  |
| HousingDensity | 0.540 | -0.233 | 0.282 | 0.758 |  |
| Standard deviation | 1.816 | 0.835 | 0.067 | 0.011 |  |
| Proportion of variance | 0.824 | 0.175 | 0.001 | 0.000 |  |
| Cumulative proportion | 0.824 | 0.999 | 1.000 | 1.000 |  |
|  |  |  |  |  |  |
| **Habitat PCA** | | | | | |
| **Parameter** | **PC1** | **PC2** | **PC3** | **PC4** | **PC5** |
| TidalMarshAcres | -0.624 | -0.087 | 0.131 | -0.036 | -0.587 |
| PctTidalMarsh | -0.441 | 0.376 | 0.047 | 0.598 | -0.069 |
| PctUrbanHabitat | -0.247 | -0.265 | -0.735 | -0.356 | -0.182 |
| PctCropland | -0.322 | -0.241 | 0.618 | -0.493 | 0.198 |
| PctPasture | 0.400 | -0.561 | 0.216 | 0.305 | -0.551 |
| PctOriginalTidalMarsh | 0.302 | 0.639 | 0.109 | -0.423 | -0.524 |
| Standard deviation | 1.575 | 1.221 | 1.085 | 0.922 | 0.000 |
| Proportion of variance | 0.414 | 0.249 | 0.196 | 0.142 | 0.000 |
| Cumulative proportion | 0.414 | 0.662 | 0.858 | 1.000 | 1.000 |

**Supplemental Table S6:** AIC results comparing the influence of PC1 and PC2 for climate, habitat, and urbanization, each parameter independently, and Fst on bill surface area.

| **Model** | **K** | **AICc** | **∆AICc** | **AICcWt** | **Cum.Wt** | **LL** |
| --- | --- | --- | --- | --- | --- | --- |
| ClimPC2 | 3 | 35.58 | 0 | 0.36 | 0.36 | -2.79 |
| Tmin | 3 | 36.72 | 1.14 | 0.20 | 0.56 | -3.36 |
| PctPasture | 3 | 38.67 | 3.09 | 0.08 | 0.64 | -4.33 |
| SampleSalinity | 3 | 39.35 | 3.77 | 0.05 | 0.69 | -4.68 |
| TDmean | 3 | 39.73 | 4.15 | 0.04 | 0.74 | -4.87 |
| Tmax | 3 | 40.83 | 5.25 | 0.03 | 0.76 | -5.42 |
| HabitatPC2 | 3 | 41.18 | 5.60 | 0.02 | 0.79 | -5.59 |
| PctCropland | 3 | 41.23 | 5.64 | 0.02 | 0.81 | -5.61 |
| Fst_interior | 3 | 41.54 | 5.96 | 0.02 | 0.82 | -5.77 |
| PctOriginalTidalMarsh | 3 | 41.60 | 6.02 | 0.02 | 0.84 | -5.80 |
| HabitatPC1 | 3 | 41.97 | 6.38 | 0.01 | 0.86 | -5.98 |
| TidalMarshAcres | 3 | 41.97 | 6.39 | 0.01 | 0.87 | -5.99 |
| VPDmax | 3 | 42.17 | 6.59 | 0.01 | 0.88 | -6.09 |
| ClimPC1 | 3 | 42.24 | 6.66 | 0.01 | 0.90 | -6.12 |
| CurrentPop | 3 | 42.70 | 7.11 | 0.01 | 0.91 | -6.35 |
| VPDmin | 3 | 42.71 | 7.12 | 0.01 | 0.92 | -6.35 |
| PctTidalMarsh | 3 | 42.77 | 7.19 | 0.01 | 0.93 | -6.39 |
| HumanPC2 | 3 | 42.83 | 7.24 | 0.01 | 0.94 | -6.41 |
| HousingDensity | 3 | 42.85 | 7.27 | 0.01 | 0.95 | -6.42 |
| HumanPC1 | 3 | 42.89 | 7.31 | 0.01 | 0.96 | -6.45 |
| HumanPopDensity | 3 | 42.89 | 7.31 | 0.01 | 0.97 | -6.45 |
| Fst_beldingi | 3 | 43.01 | 7.42 | 0.01 | 0.97 | -6.50 |
| PctUrbanHabitat | 3 | 43.06 | 7.47 | 0.01 | 0.98 | -6.53 |
| PctPopulation | 3 | 43.06 | 7.47 | 0.01 | 0.99 | -6.53 |
| Prec | 3 | 43.06 | 7.48 | 0.01 | 1 | -6.53 |

**Supplemental Table S7:** AIC results comparing the influence of PC1 and PC2 for climate, habitat, and urbanization, each parameter independently, and Fst on different bill dimensions. For bill length and bill width only models within a ∆AICc<5 are shown and for bill depth models within a ∆AICc<10 are shown.

| **Bill Length** | | | | | | |
| --- | --- | --- | --- | --- | --- | --- |
| **Model** | **K** | **AICc** | **∆AICc** | **AICcWt** | **Cum.Wt** | **LL** |
| ClimPC2 | 3 | 33.83 | 0 | 0.31 | 0.31 | -1.91 |
| HabitatPC1 | 3 | 33.89 | 0.06 | 0.30 | 0.60 | -1.94 |
| TidalMarshAcresCurrent | 3 | 35.77 | 1.95 | 0.12 | 0.72 | -2.89 |
| PctPasture | 3 | 36.62 | 2.79 | 0.08 | 0.80 | -3.31 |
| Tmax | 3 | 37.29 | 3.47 | 0.05 | 0.85 | -3.65 |
| VPDmax | 3 | 37.78 | 3.95 | 0.04 | 0.89 | -3.89 |
| Tmin | 3 | 38.07 | 4.24 | 0.04 | 0.93 | -4.03 |
| **Bill Width** | | | | | | |
| **Model** | **K** | **AICc** | **∆AICc** | **AICcWt** | **Cum.Wt** | **LL** |
| Fst_beldingi | 3 | 35.02 | 0 | 0.35 | 0.35 | -2.51 |
| HousingDensity | 3 | 37.17 | 2.15 | 0.12 | 0.47 | -3.58 |
| HumanPopDensity | 3 | 37.60 | 2.58 | 0.10 | 0.57 | -3.80 |
| CurrentPop | 3 | 38.25 | 3.23 | 0.07 | 0.63 | -4.12 |
| Prec | 3 | 38.27 | 3.26 | 0.07 | 0.70 | -4.14 |
| HabitatPC2 | 3 | 39.05 | 4.03 | 0.05 | 0.75 | -4.52 |
| HumanPC1 | 3 | 39.55 | 5.53 | 0.04 | 0.79 | -4.77 |
| PctUrbanHabitat | 3 | 39.57 | 4.55 | 0.04 | 0.82 | -4.78 |
| HumanPC2 | 3 | 40.06 | 5.04 | 0.03 | 0.85 | -5.03 |
| **Bill Depth** | | | | | | |
| **Model** | **K** | **AICc** | **∆AICc** | **AICcWt** | **Cum.Wt** | **LL** |
| Fst_interior | 3 | 30.34 | 0 | 0.46 | 0.46 | -0.17 |
| VPDmin | 3 | 31.09 | 0.76 | 0.32 | 0.78 | -0.55 |
| ClimPC1 | 3 | 32.47 | 2.13 | 0.16 | 0.94 | -1.23 |
| PctTidalMarsh | 3 | 37.38 | 7.05 | 0.01 | 0.95 | -3.69 |
| TDmean | 3 | 37.71 | 7.38 | 0.01 | 0.97 | -3.86 |
| Prec | 3 | 38.53 | 8.16 | 0.01 | 0.97 | -4.25 |
| VPDmax | 3 | 39.60 | 9.26 | 0 | 0.98 | -4.80 |
| SampleSalinity | 3 | 39.66 | 9.33 | 0 | 0.98 | -4.83 |

**Supplemental Table S8:** Predicted water savings between historic and modern samples of Savannah Sparrow given observed change in bill surface area (Fig. 7). The percent of daily water budget was estimated from calculations of expected amount of daily water loss given the mean mass of Savannah Sparrows in the dataset (17.14g).

| **Locality** | **Change in mean bill surface area (% change)** | **Predicted change in TEWL (mg^-1^ g^-1^ h^-1^)** | **Predicted change in TEWL (mg^-1^ h^-1^)** | **Percent of daily water budget** |
| --- | --- | --- | --- | --- |
| Humboldt Bay | -2.36 (3.86%) | 0.46 | 7.88 | -8.3% |
| Suisun Bay | 0.83 (1.43%) | -0.16 | -2.74 | 2.8% |
| San Pablo Bay | 3.07 (5.42%) | -0.60 | -10.28 | 10.8% |
| San Francisco Bay | 1.06 (1.82%) | -0.21 | -3.60 | 3.8% |
| Morro Bay | 4.55 (7.37%) | -0.89 | -15.43 | 16.2% |
